# Interdisciplinary pain program participants with high catastrophizing scores improve function utilizing enriched therapeutic encounters and integrative health techniques: a retrospective study

**DOI:** 10.3389/fpsyg.2024.1448117

**Published:** 2024-09-13

**Authors:** Ariana Vora, Eve Kennedy-Spaien, Sarah Gray, Anayali Maria Estudillo-Guerra, Gabriele Phillips, Ines Mesia-Toledo, Mel Glenn, Bridget S. Chin, Leon Morales-Quezada

**Affiliations:** ^1^Spaulding Integrative Health Initiative, Spaulding Rehabilitation Hospital, Boston, MA, United States; ^2^Department of Physical Medicine & Rehabilitation, Spaulding Rehabilitation Hospital, Boston, MA, United States; ^3^Harvard Medical School, Boston, MA, United States; ^4^Pain and Functional Restoration Program, Spaulding Rehabilitation Hospital, Medford, MA, United States; ^5^Department of Occupational Therapy, Spaulding Rehabilitation Hospital, Boston, MA, United States; ^6^Department of Psychiatry, Massachusetts General Hospital, Boston, MA, United States; ^7^Spaulding Research Institute, Spaulding Rehabilitation Hospital Network, Boston, MA, United States; ^8^Department of Occupational Therapy, MGH Institute of Health Professions, Boston, MA, United States; ^9^College of Human Medicine, Michigan State University, East Lansing, MI, United States

**Keywords:** pain catastrophizing, functional outcomes, chronic pain, interdisciplinary/multidisciplinary pain management programs, integrative health, complementary medicine, pain neuroscience education, enriched therapeutic encounters

## Abstract

**Introduction:**

*Pain catastrophizing* describes helplessness, rumination, and magnification of a pain experience. High pain catastrophizing is an independent risk factor for disability, pain severity, inadequate treatment response, chronicity, and opioid misuse. Interdisciplinary pain programs (IPPs) are beneficial and cost-effective for individuals with chronic pain, but their functional impact on individuals with high pain catastrophizing is not well established. The emerging field of placebo studies suggests that patient-provider relationships, positive treatment expectations, and sociobiologically informed care trigger physiological responses that may enhance therapeutic interventions.

**Methods:**

In this retrospective observational cohort study, we compared admission and discharge data for 428 adults with high-impact chronic pain (mean 8.5 years) who completed the Spaulding-Medford Functional Restoration Program (FRP). The interdisciplinary FRP team of physiatrists, behavioral health clinicians, physical therapists, and occupational therapists specializes in evidenced-based conventional rehabilitation, integrative health, and pain psychoeducation via enriched therapeutic encounters, fostering collaboration, validation, trust, self-efficacy, and positive expectations. Clinical outcome measures included the Canadian Occupational Performance Measure (COPM) assessing functional performance (COPM-PS) and satisfaction with function (COPM-SS), the Pain Numeric Rating Scale (NRS), the Pain Catastrophizing Scale (PCS), and the Patient Health Questionnaire-9 (PHQ-9).

**Results:**

FRP participants with clinically elevated catastrophizing at baseline (PCS ≥30, mean PCS 39) achieved statistically significant improvements in function (mean delta -2.09, CHI2 = 15.56, *p* < 0.001), satisfaction with function (COPM-SS mean delta -2.50, CHI2 = 7.42, *p* = 0.007), pain (NRS mean delta 2.7), mood (PHQ-9 mean delta 1.87, *p* = 0.002), and catastrophizing (PCS mean delta 4.16, *p* < 0.001). Subgroup analysis revealed racial disparities in pain scores, and exploratory analysis showed a trend toward reducing opiate consumption.

**Discussion:**

Despite the known association of adverse outcomes with high catastrophizing, FRP participation was associated with increased productive engagement, reduced pain, reduced maladaptive thought processes, and improved mood. Although causation and efficacy cannot be established from a retrospective design, this is the first study to identify functional improvement in patients with high-impact chronic pain and clinically relevant high pain catastrophizing who participate in an IPP combining conventional and complementary rehabilitation with psychoeducation. These enriched therapeutic encounters may enhance the treatment process by promoting trust, empathy, collaboration, and beneficial reframing of patients’ experiences, expectations, and goals.

## 1 Introduction

### 1.1 Scope and context of the problem

Pain affects 145 million U.S. adults, with 50.2 million (20.5%) experiencing pain most days (Ehde et al., 2014; Toth et al., 2014; Yong et al., 2022). Health care expenses and lost productivity due to chronic pain exceed $560 billion annually (Ehde et al., 2014), and 43 per 100 Americans receive opioid prescriptions (Townsend et al., 2008) Despite this, many individuals with high-impact chronic pain receive minimal symptom relief or functional improvement. High-impact chronic pain (HICP) is ≥ 6 months of persistent pain that “substantially restricts work, social, and self-care activities” (Dahlhamer et al., 2018). Eighty-three percent of individuals living with HICP become unemployed and have difficulty with basic activities of daily living (ADLs) (IPRCC, 2020). Individuals with HICP have more comorbid health conditions, cognitive impairment, mental health issues, and opioid use than those with low-or moderate-impact chronic pain. HICP is associated with increased healthcare utilization, including emergency room visits, specialty appointments, and procedures, often without symptomatic or functional improvement (Pitcher et al., 2019). Failed treatment attempts reinforce the belief that nothing will improve, contributing to functional impairment (Bingel, 2020; Finan et al., 2022; Lin et al., 2017), financial stress (Yong et al., 2022), hopelessness, and suicide risk (Tang, 2006).

*Pain catastrophizing* is a negative cognitive–affective response to anticipated or actual pain (Quartana et al., 2009), initially described by Ellis (1962). Although the term has become controversial in recent years, the authors have chosen to use it here to maintain consistency with the body of research referenced. Pain catastrophizing is an independent risk factor for disability, pain chronicity, intensity, and opioid misuse (Angst et al., 2022; Leung, 2012; Martel et al., 2013; Wertli et al., 2014). It is associated with hyperexcitability in brain regions mediating pain anticipation (medial frontal cortex, cerebellum), attention to pain (anterior cingulate cortex, dorsolateral prefrontal cortex), and emotion (amygdala), leading to aberrant plasticity, central sensitization and nociplastic pain (Gracely et al., 2004). Pharmacologic and psychosocial treatments for chronic pain are less effective in populations with elevated pain catastrophizing (Mankovsky et al., 2012; Toth et al., 2014). Furthermore, 70 percent of individuals scoring higher than 30 on the 52-point Pain Catastrophizing Scale (PCS) are unemployed a year after symptom onset and identify as “totally disabled” in their occupation; and 66 percent score higher than 16 (moderately depressed) on the Beck Depression Inventory-II (Schutt et al., 2016; Weiss et al., 2013), which is associated with higher pain intensity perception (Turk, n.d.; Turk and Swanson, 2019).

### 1.2 Background of interdisciplinary pain programs, and Functional Restoration Program

Interdisciplinary pain management programs (IPPs) are integrated teams, usually including physicians, physical therapy, and behavioral health, that address maladaptive cognitions including pain catastrophizing and foster positive treatment expectations associated with functional improvement (Craner et al., 2016; Myers et al., 2008; Sullivan et al., 1998). IPPs result in superior quality of life and functional outcomes including return to work, and they are more effective and cost-efficient than medical treatment or physical therapy alone (Casey et al., 2020; Murphy et al., 2011; Pain Management Best Practices Inter-Agency Task Force Report, n.d.; Townsend et al., 2008; Turk, n.d.). However, among participants with elevated pain catastrophizing scores (PCS), Moore et al. (2016) found decreased maintenance of IPP treatment gains. Scott et al. (2014) found that pre-treatment PCS scores >24 correlated with poor clinical outcomes, and post-treatment PCS scores ≥14 correlated with not returning to work. The significance of these findings is underscored by a recent large-scale study indicating that among 13,000 individuals with chronic pain, the average PCS score was 29.8 (Nicholas et al., 2019). Many studies exclude participants based on psychological factors including high pain catastrophizing (Salmasi et al., 2022), and this exposes a critical gap in the literature on evidence-based strategies to enhance function for individuals with high pain catastrophizing.

The Functional Restoration Program (FRP) is an outpatient IPP designed to help individuals with high impact chronic pain to understand the complex neurophysiologic, cognitive, psychosocial, and emotional factors that impact pain; develop self-efficacy in managing pain; improve function in all domains of daily living (self-care, home, work, leisure, and community); and enhance quality of life. The interdisciplinary FRP team consists of experienced physiatry, behavioral health, occupational therapy, and physical therapy clinicians providing evidence-based, individualized outpatient care via group and individual sessions. Participation is covered by insurance, except for acupuncture and massage which are not core FRP treatments but available in the clinic on a self-pay basis.

FRP clinicians combine conventional rehabilitation with evidence-based integrative health practices including meditation, yoga, and tai chi, which have previously demonstrated efficacy for chronic pain (Hilton et al., 2017; Holtzman and Beggs, 2013; Kizhakkeveettil et al., 2014; Lin et al., 2017; Peng, 2012). FRP participants also receive biofeedback training, which is a mind–body technique for self-regulating heart rate, breathing, muscle guarding, and other signs and symptoms of stress that has been shown to reduce pain, enhance coping, reduce muscle tension, and relieve depression in people with various musculoskeletal disorders (Kent et al., 2015; Nestoriuc et al., 2008). In people with low back pain, combining complementary therapies with conventional medical care or exercise is more effective than conventional care or exercise alone (Holtzman and Beggs, 2013; Kaptchuk et al., 2020; Kizhakkeveettil et al., 2014; Williams et al., 2020). Numerous studies support the use of acupuncture and acupressure in chronic pain management, especially for osteoarthritis, headache, low back pain (Godley and Smith, 2020) and other musculoskeletal pain (Kizhakkeveettil et al., 2014; Qaseem et al., 2020; Yeh et al., 2022).

### 1.3 Placebo, nocebo, enriched therapeutic encounters, and contextual factors

Pain is a complex interaction of physical, mental, and emotional experiences. Contemporary understanding emphasizes the roles of perception and experience in the creation, intensification, and perpetuation of pain, underscoring the potential of leveraging placebo effects while decreasing nocebo responses. Placebo research has demonstrated that classical conditioning and positive treatment expectations generate positive clinical outcomes. In fact, treatment response expectancies predict nonvolitional responses to events (Kirsch, 2018), including expected pain reduction. Pain reduction driven by positive expectations, often termed “placebo analgesia,” can activate neural pathways that modulate pain perception through the orbitofrontal and prefrontal cortex, cingulate, and periaqueductal gray (Kirsch, 1985; Sawamoto et al., 2000). This process is blocked by naloxone, a *μ*-opioid receptor agonist that physiologically inhibits reactivity to noxious stimuli in the anterior cingulate, insula, and thalamus (Atlas, 2021). Research in the fields of placebo and nocebo effects demonstrates that embodied psycho-neurobiological responses modulate symptoms. This underscores a key aspect of FRP participation: Addressing the meaning of the pain.

Contextual factors are defined by Cook et al. (2023) as “perceived cues that affect both the patient and practitioner and can arise from previous experiences and immediate dynamics within the encounter, or a combination of both;” and these extend to the dynamic between patient and practitioner and the health care environment. Enriched therapeutic encounters facilitate contextual factors that trigger neurobiological, perceptual, and cognitive mechanisms increasing the quality of therapeutic outcomes. Positive contextual factors encourage 1) empathy; 2) active listening; 3) collaborative goal setting; 4) exploration and reflection; 5) integration of evidence-based therapeutic techniques; 6) creative expression and experiential activities; and 7) cultural sensitivity and inclusivity (Cook et al., 2023; Sherriff et al., 2022; Testa and Rossettini, 2016). They also relieve pain by producing placebo-like effects, while reducing pain-aggravating nocebo-like effects (Testa and Rossettini, 2016).

Individuals with persistent pain often seek out or are referred to a multitude of specialists, resulting in a higher probability of exposure to conflicting advice and to language that reinforces nocebo beliefs that they are damaged or injured (Rossettini et al., 2022). These negative contextual factors are “danger messages,” as Mosley and Butler (2017) describe in pain neuroscience literature. Through the FRP, participants explore their beliefs regarding their pain and prognosis, and they receive education on neuroplasticity to enhance their understanding and promote positive treatment expectations. Concurrently, through experiential learning, treatment is designed to change predictive coding and enhance interoception. This top-down and bottom-up approach is designed to decrease pain catastrophizing and improve function, and facilitate psychoeducation, personal growth, healing, and positive change through interdisciplinary, collaborative, supportive, and empowering therapeutic relationships (Kaptchuk et al., 2020). Furthermore, observing peers experiencing positive treatment outcomes has been shown to enhance functional change and increase pain analgesia (Schwartz et al., 2022).

### 1.4 Objectives

The primary aim of this investigation is to evaluate functional outcomes and pain levels in individuals with high pain catastrophizing before and after Functional Restoration Program (FRP) participation, comparing low and high catastrophizing subgroups for differences in function, satisfaction with function, and pain. The secondary aim is to evaluate changes in mood and pain catastrophizing following FRP participation. While staff did not systemically collect data on opiate use due to the nonpharmacologic nature of this program, we also aimed to perform an exploratory analysis of the available opiate data. We hypothesized that both groups would demonstrate significant improvement in function and mood, and that this improvement would be significant, but not as pronounced in high-catastrophizing subgroup. As a retrospective chart review, we could not establish efficacy or causation, so we hypothesized that these would be measured through positive associations. Since the program emphasizes self-management rather than pain reduction and since we did not employ an opioid reduction protocol, we did not anticipate changes in pain rating or opioid use.

## 2 Materials and methods

### 2.1 Study design

We performed a retrospective review on 428 adults who completed the Functional Restoration Program (FRP), an interdisciplinary pain program at Spaulding Rehabilitation Network’s Outpatient Center in Medford, Massachusetts, from 2016 until March 2020 when in-person outpatient care was replaced with a virtual platform during the coronavirus pandemic. Outcomes were collected as part of the FRP’s standard continuous quality improvement process. This study was approved by our Institutional Review Board which waived informed consent for this retrospective chart review. Being a medical record review, the study was not pre-registered. All aspects of the study were in accordance with the Helsinki Declaration (World Medical Association, 2000), and reporting was based on the Strengthening the Reporting of Observational Studies in Epidemiology guidelines https://www.strobe-statement.org (checklist available in the supplement materials) (Cuschieri, 2019). Sources of potential bias were addressed to ensure validity and reliability of the study’s findings. We followed STROBE guidelines of comprehensive and transparent reporting of the study design, methods, and results to enhance the credibility and generalizability of the findings.

### 2.2 Inclusion/exclusion criteria and screening

This study utilized the same inclusion criteria, exclusion criteria and screening process that are used clinically for FRP enrollment. Inclusion criteria are based on the definition of high-impact chronic pain (COPM, n.d.), and exclusion criteria are based on clinical judgment including safety needs, ability to commit to the program requirements, and openness to an active, self-care approach.

#### 2.2.1 Inclusion criteria

(1) Age ≥ 18 years; (2) Pain duration ≥6 months; (3) Failure of at least one traditional treatment approach such as medication, injections, or physical therapy; (4) Functional limitation in at least 2 activities of daily living (ADLs) and instrumental activities of daily living (IADLs) due to pain.

#### 2.2.2 Exclusion criteria

(1) Medical diagnoses that preclude safe participation in the program (e.g., recent myocardial infarction, surgery, or unhealed acute injury); (2) Acute psychiatric issues that would preclude safe ability to participate in the program and require more intensive behavioral health support than our program provides (e.g., recent suicide attempt or active psychosis); (3) Inability to consistently attend treatment sessions, as the FRP has strict attendance requirements; (4) Patients who verbalize apparent lack of interest in a rehabilitation approach and are exclusively focused on further diagnostics, surgeries, or opioids.

Screening of prospective participants begins with a thorough evaluation by a Physical Medicine & Rehabilitation physician, also known as a physiatrist, to determine whether immediate diagnostic or medical/surgical interventions are indicated and whether FRP participation is safe from a musculoskeletal and medical standpoint. Once the physiatrist recommends the patient for the program, the remaining team members evaluate and determine whether individual visits or combined group and individual visits would best meet the participant’s unique needs based on their clinical judgment and participant input regarding goals, time constraints, and transportation. For example, FRP staff may recommend individual rather than group treatment for individuals with significant cognitive deficits, sensory sensitivities, or safety issues requiring high levels of supervision.

### 2.3 Intervention

#### 2.3.1 Unified program interventions (cross-discipline)

FRP goals are to improve function, develop skills to self-manage pain, and provide pain neuroscience education (Louw et al., 2016) for individuals with HICP. At enrollment, each participant signs a treatment agreement outlining program expectations (e.g., attendance, home program, duration of therapy), reinforcing the program’s goals to improve function and to manage rather than eliminate pain. Participants then list their functional goals in collaboration with their team. Interventions include strength training, aerobic conditioning, work/functional simulation, coping strategies such as pacing, and cognitive-behavioral strategies to address functional and biopsychosocial aspects of pain (Table 1). The team meets weekly to discuss each participant and modify the care plan as needed to promote functional progress.

**Table 1:** Treatments that each FRP participant receives.

Treatment
Acceptance and Commitment Therapy
Acupuncture^a^, self-acupressure
Biofeedback (sEMG, HRV, temperature)
Body Mechanics training for ADLs/IADLs
Cardiovascular conditioning
Cognitive Behavioral Therapy
Core Movement Integration +/or Feldenkrais
Discharge planning
Energy conservation
Flare-up management
Imagery (graded motor, guided)
Joint protection training
Massage^a^, self-massage
Mindfulness and guided imagery
Pacing
Pain and neuroscience education
Strengthening
Stretching
Tai Chi
Thermal modalities (ice, heat, contrast baths)
Yoga

a Acupuncture and Massage are available on self-pay basis.

At the first meeting, the Physical Medicine & Rehabilitation physician provides education on pain neuroscience, peripheral and central sensitization, and the importance of self-management. Starting early in treatment, clinicians from each discipline on the team regularly provide pain neuroscience education to reduce fear of movement (Blickenstaff and Pearson, 2016), using language distinguishing pain versus injury and utilizing a cognitive-behavioral framework that reinforces function-enhancing thoughts and behaviors (Urits et al., 2019). Through this coordinated education, participants learn to better understand their pain, interpret sensations, self-manage symptoms, and discover strategies for safely and confidently re-engaging in their important life roles.

**Figure 1:**
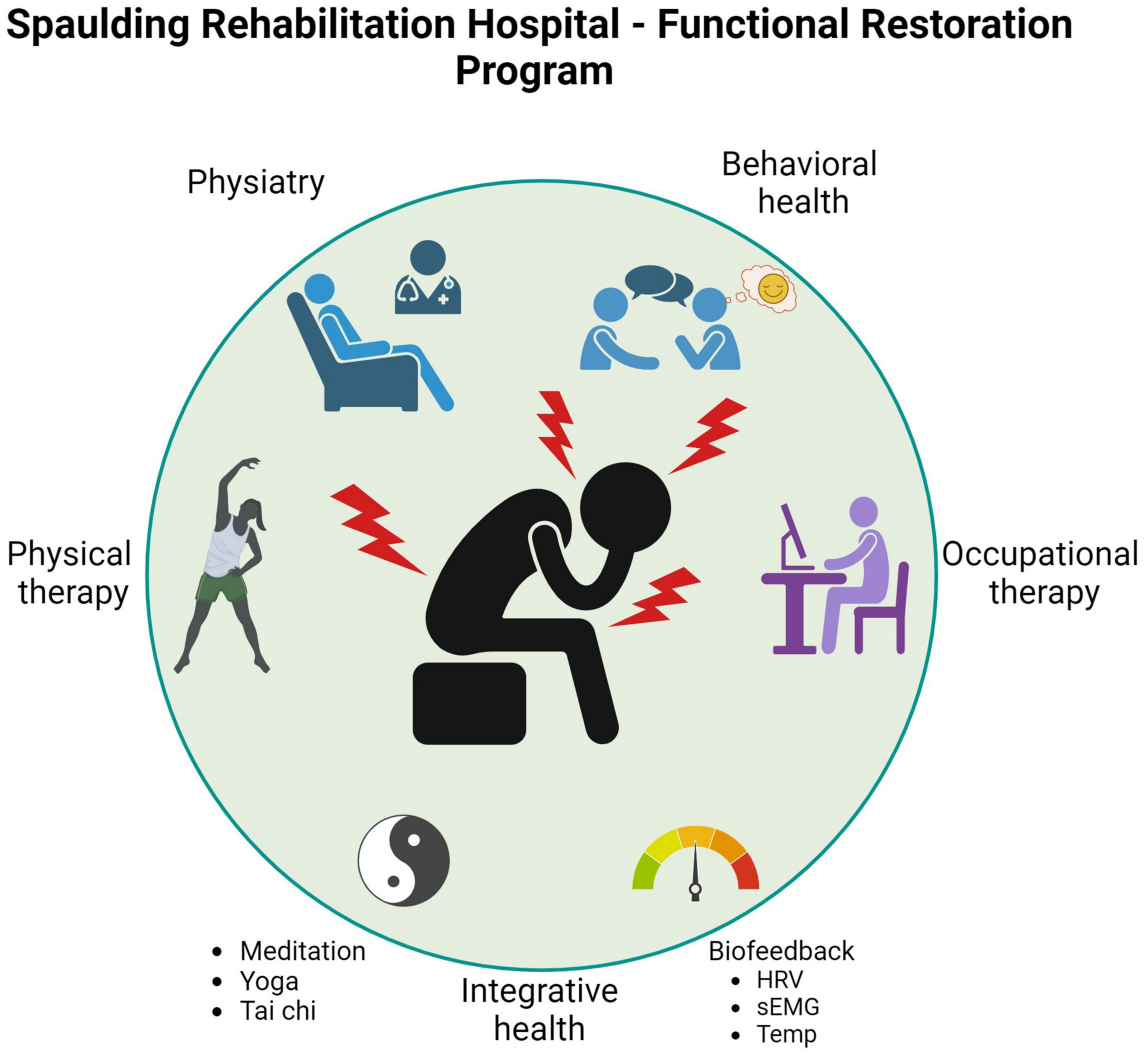
Interdisciplinary biopsychosocial approach of Functional Restoration Program.

Using expert clinical judgment, the FRP team creates an individualized treatment plan for interdisciplinary rehabilitation with enriched therapeutic encounters based on each participant’s unique needs including goals, ability, and availability. Between group and individual formats, participants receive 16 occupational and physical therapy sessions and 2–8 behavioral health sessions over an average period of 8 weeks. Treatment is intensive as participants work with physical therapy, occupational therapy, and behavioral health clinicians for consecutive sessions of at least 45 min each, 1–2 days per week, and follow home programs on days when they are not in the clinic. Prior to completing the program, participants work with their team to develop an individualized discharge plan that includes instructions for returning to structured activity (e.g., work, school, or volunteering) and strategies for recognizing and managing pain flare-ups (Figure 1).

#### 2.3.2 Discipline-specific interventions

Each discipline approaches integrative health modalities from its own frame of reference, aiming to enhance function and quality of life FRP participants receive three or more biofeedback sessions and weekly instruction on tai chi, yoga, and mindfulness techniques. Aiming to recognize and lower signs of excessive physiological arousal, reduce muscle guarding, and improve movement patterns. Additionally, FRP staff train participants in all the techniques listed in Table 1. Home programs include at least one integrative health practice daily to reinforce skill training and enhance self-efficacy.

##### 2.3.2.1 Physical therapy

Physical therapy performed within the context of an interdisciplinary program (Davin et al., 2019) and combined with pain neuroscience education has been found to be more effective than physical therapy alone (Siddall et al., 2022; Wood and Hendrick, 2019). FRP physical therapists (PT) specialize in treating patients with chronic pain and work on reducing kinesiophobia and fear-avoidance behavior, enhancing strength and postural stabilization, improving alignment, and reducing antalgic dysfunctional patterns. Participants enter the FRP having trialed PT previously and often are skeptical due to unsuccessful past attempts. Individuals with widespread pain conditions often suffer exercise induced hyperalgesia instead of hypoalgesia (Vaegter and Jones, 2020), which further promotes fear avoidance. PTs begin with pain neuroscience education, explaining the impact of movement and exercise in mediating central sensitization, and promoting strength, flexibility, and physical tolerance. Utilizing a top-down approach, they address the inhibitory and facilitatory pain mechanisms at the level of the periphery and centrally. PTs work with the patient to develop a customized program of individualized stretching, strengthening and cardiovascular conditioning. The program is graded and modified to ensure successful completion and minimize pain exacerbations. They instruct participants in proactive use of self-management strategies, including self-massage and thermal pain control modalities such as heat or cold pack application and ice massage. This enhances self-efficacy, as participants are encouraged to experiment and discover modalities that are most beneficial for them (Rakel and Barr, 2003).

In addition to more traditional physical therapy interventions, FRP PTs integrate Feldenkrais/Awareness Through Movement and core movement integration into each participants program. This is performed individually and in a group environment. These mindfully performed, gentle movements have been shown to improve quality of life, enhance interoceptive awareness and reduce disability (Ahmadi et al., 2020; Berland et al., 2022). These techniques provide opportunities to experience movement without pain elevations, increase body awareness of non-pain stimuli, facilitate efficient movement patterns, and reduce guarded movement patterns that inhibit range of motion, flexibility, and quality of movement.

##### 2.3.2.2 Occupational therapy

Occupational therapists (OTs) help individuals with chronic pain find comfortable ways to engage in meaningful, valued life activities (Lagueux et al., 2018; Lagueux et al., 2023). While performing daily tasks, OTs help FRP participants learn new methods of activity performance. Strategies include body mechanics, ergonomics, and joint protection to enhance biomechanical advantage; pacing and energy conservation to reduce fatigue and minimize flare-ups; and incorporation of pain control tools within the home, work, and community (Breeden and Rowe, 2022). This experiential learning allows participants to alter their pain expectations, which can help reduce fear avoidance patterns (Finan et al., 2022; Janssens et al., 2019).

FRP OTs utilize yoga and tai chi to enhance proprioception and quality of movement, decrease physiologic arousal, and offer an alternative to opioids for pain management (Büssing et al., 2012; Kong et al., 2016; Peng, 2012; Yan et al., 2013). Participants perform instrumental activities of daily living such as vacuuming, raking, and cooking while incorporating fluid, rhythmic upper and lower extremity tai chi-based movement patterns and weight shifts that improve balance (Howe et al., 2011). Using adaptive hatha yoga, OTs teach individualized positions that improve range of motion, promote core stability, and reduce muscle tension (Büssing et al., 2012; Marshall et al., 2022).

Biofeedback has been shown to reduce pain, improve movement patterns, enhance QOL and function (Kent et al., 2015), improve coping, reduce muscle tension, and reduce depressive symptoms (Nestoriuc et al., 2008). As patients perform goal–oriented tasks, such as lifting or computer work, OTs help them optimize motor patterns utilizing immediate visual and auditory feedback via surface electromyography. In this operant conditioning paradigm, participants activate targeted muscle groups while inhibiting muscles associated with maladaptive activation and inhibition patterns (Kent et al., 2015; Neblett et al., 2003; Peper et al., 2003).

##### 2.3.2.3 Behavioral health

Behavioral Health treatment includes structured Cognitive Behavioral Therapy, Acceptance and Commitment Therapy, and motivational interviewing to promote changes in thoughts, beliefs, and behaviors in alignment with FRP participants’ values, functional goals, and mood-based goals. Tailoring treatment to individual needs, FRP behavioral health providers utilize strategies such as cognitive restructuring, journaling, thought logs, value identification, optimizing sleep hygiene, self-compassion experiential exercises, and pain neuroscience psychoeducation. These techniques improve mood, quality of life, and utilization of coping skills, reducing stress and nervous system stimulation that can, in turn, exacerbate pain (Bernardy et al., 2013; Giles, 2014; Thorn, 2004; Williams et al., 2020). Participants decrease psychophysiological arousal and somatic focus through mindfulness techniques, guided imagery, progressive muscle relaxation, and biofeedback measuring heart rate variability (Reneau, 2020), galvanic skin response or distal hand temperature to recognize and self-regulate physiological processes.

### 2.4 Outcome measures

The following measures were completed at initial evaluation and at discharge.

#### 2.4.1 Canadian occupational performance measure

The Canadian Occupational Performance Measure (COPM) is a patient-centered functional outcome tool that has been validated across multiple populations, including individuals with chronic pain, and demonstrates sensitivity to change (Carpenter et al., 2001; Persson et al., 2013). Participants identify their top five functional priorities and then rate them from 1 to 10 on two subscales, generating a performance score (COPM-PS) and a satisfaction-with-performance score (COPM-SS). Performance and satisfaction scores are calculated by averaging the five scores. Clinical significance has previously been defined in the literature as a positive change score of ≥2.0 for performance and satisfaction (COPM, n.d.).

#### 2.4.2 Pain Catastrophizing Scale

The Pain Catastrophizing Scale (PCS) is a commonly utilized self-report scale examining patterns of thinking and perception that may contribute to fear-based avoidance of activity and related maladaptive behaviors that can perpetuate and intensify the impact of chronic pain (Sullivan et al., 1995; Weiss et al., 2013). It also demonstrates sensitivity to change and can be used to assess treatment outcomes (Anamkath et al., 2018). Participants rate 13 statements between 0 and 4, with 0 as “never” and 4 as “always.” The total possible score ranges from 0 to 52, with a higher number indicating more frequent and severe catastrophic thoughts. High PCS scores have been shown to predict worsening mood and function, higher rates of disability, and higher pain ratings (Thorn, 2004). Participants in the 75th percentile (total score above 30) are at the highest risk for chronicity (Craner et al., 2016). The cutoff point at which PCS scores are thought to predict adverse outcomes varies widely in the literature, with some studies setting it as low as 15 or 24, and others as high as >38 (Craner et al., 2016; Scott et al., 2014). We selected a cutoff of 30 because it is the benchmark determined by the authors of the PCS, Sullivan and Bishop, and represents the 75th percentile of distribution when researched in a clinical setting (Sullivan et al., 1995).

#### 2.4.3 Numeric Pain Rating Scale

The Numeric Pain Rating Scale (NRS) is a validated, well-established rating scale that evaluates pain intensity (Jensen et al., 1999). To compensate for the variability of day-to-day pain levels, participants verbally rate their best (NRS-low) and worst (NRS-high) pain levels over the last week on a scale from 0 to 10 at FRP admission and discharge; 0 denotes “no pain” and 10 denotes” worst possible pain.” Prior research has established a change of 1.5 to 2.5 as clinically significant (Modarresi et al., 2021).

#### 2.4.4 Patient health questionnaire

Organizations such as the Initiative on Methods, Measurement, and Pain Assessment in Clinical Trials (IMMPACT, n.d.) workgroup recommend screening for depression/emotional function as one of the six core domains for a comprehensive assessment of chronic pain. The Patient Health Questionnaire (PHQ) is a widely utilized self-report screening measure developed from a primary care diagnostic tool, the PRIME-MD (Spitzer et al., 1999), to screen for twelve mental health disorders. It was simplified into sub-screening tools for focused symptoms or brief screening items, such as the PHQ-2 or PHQ-4, with the numbers designating the number of questions in the tool. We use the PHQ-9, which screens for depressive symptoms derived from the DSM-5 criteria for major depressive disorder, to screen for participants who may need further assessment and treatment of these symptoms. A high PHQ-9 score alone is insufficient to diagnose depression; a trained clinician must further assess several factors to make an official diagnosis. For these reasons, in addition to the ease of administration, free access to the scale, and the scale’s translation into other languages, FRP participants complete the PHQ-9 in addition to meeting with the FRP behavioral health clinician for an assessment as part of the intake process.

### 2.5 Statistical methods

Statistical analysis was performed using STATA v.13.1 software (STATA Corp, College Station, Texas). The statistical significance level was defined with two-tailed *p* < 0.05. Confidence intervals were defined at the 95% confidence level. Descriptive statistics (mean, frequency, range, and percentage) were used to describe socio-demographic variables. We further differentiated PCS scores by race and performed a multivariate analysis using linear regression to assess for mean differences.

We performed univariate analysis using paired t-tests applied over the difference between clinical characteristics at baseline and post-intervention. Comparing within-group changes in means, we applied the paired Student’s *t*-test, assigning *p*-values <0.05 as statistically significant. For effect size interpretation (Cohen’s d), we assigned the following: Negligible (0–0.19), small (0.2–0.49), moderate (0.5–0.79), and large (>0.8). For the categorical outcomes of clinically significant change on COPM-PS and COPM-SS subscales, we stratified the sample based on PCS scores <30 or ≥ 30 and performed chi-square tests. We utilized a per protocol approach in our intention to treat analysis. Thus, for participants who were missing a post-FRP score in one or more of the outcome measures, data were analyzed as if there had been no change in that outcome measure from the beginning to the end of their FRP participation.

To evaluate for changes in the primary and secondary outcome measures, we utilized multivariate fixed-effect regression models to account for within-subject correlation. The dependent variables in the models represent the primary outcomes (function, satisfaction with function, and pain), and the secondary outcomes (depressive symptoms and catastrophizing scores). The independent variables were selected based on previous assumptions to evaluate for possible confounding or effect modification; we included age (continuous variable), gender, race, or ethnicity, baseline PCS scores, and the interaction of baseline catastrophizing scores with time.

## 3 Results

### 3.1 Data collection and demographics

Data were collected between 2016 and the start of the pandemic lockdown in March 2020. 428 individuals with complete baseline data graduated from the FRP program during this period. No adverse events were reported during or after FRP participation. The mean duration of high-impact chronic pain was 8.5 years, and 43 percent of participants had PCS scores ≥30. Table 2 summarizes participants’ demographic characteristics. Table 3 shows the mean differences of baseline PCS scores stratified by race/ethnicity.

**Table 2:** Demographic and clinical baseline characteristics (*n* = 428).

	*n* (%)	Mean (SD)
*Age (years)*		49.32 (14.69)
*Gender*		
Female	307(71.73)	
Male	121 (28.27)	
*Race/Ethnicity n (%)*		
White	304 (71.03)	
Black	29 (6.78)	
Latino	20 (4.67)	
Asian	9 (2.10)	
Other	40 (9.35)	
Unknown	26 (6.07)	
*NRS-high*		8.61 (1.54)
*NRS-low*		3.62 (2.22)
*COPM-PS*		3.42 (1.7)
*COPM-SS*		2.63 (1.23)
*PHQ-9*		12.38 (6.49)
*PCS*		28.8 (1189)

NRS-High, Numeric Pain Rating Scale (highest pain); NRS-Low, Numeric Pain Rating Scale (lowest pain); COPM-PS, Canadian Occupational Performance Measure–Performance Score; COPM-SS, Canadian Occupational Performance Measure–Satisfaction Score; PHQ-9, Patient Health Questionnaire-9; PCS, Pain Catastrophizing Scale.

**Table 3:** Baseline pain catastrophizing scores, differentiated by race.

Independent variable	Slope	Standard error	T-ratio	Probability
Race / Ethnicity				
Black	7.24	2.27	3.18	0.002*
Latino	4.97	2.70	1.84	0.067
Asian	−3.70	3.96	−0.94	0.35
Other	4.88	1.97	2.48	0.014*
Unknown	0.95	2.39	0.40	0.69

Constant = 26.92; R2 = 0.042; F-Ratio = 3.71; *p* = 0.0002; Standard Error of the Estimate: 11.72, *N* = 428; *symbol denotes statistical significance at < *p* = 0.05.

### 3.2 Primary outcome measures

**Figure 2:**
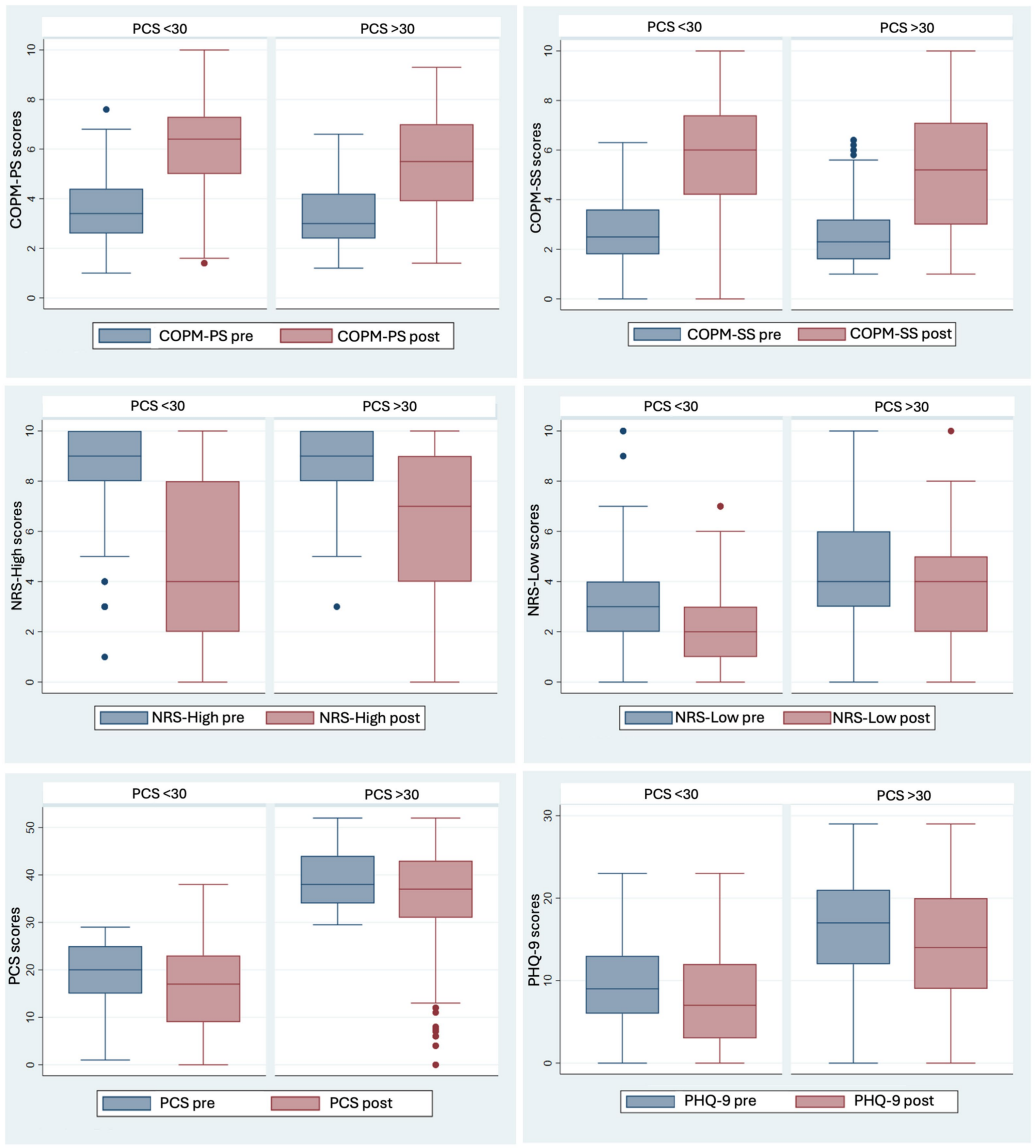
Comparison of Pre- and Post- clinical outcomes in Functional Restoration Program (FRP) with Impact of Low vs. High Baseline PCS Scores on COPM, NRS, PCS, and PHQ-9 Changes. COPM-PS, Canadian Occupational Performance Measure-Performance Score; COPM-SS, Canadian Occupational Performance Measure-Satisfaction Score; NRS-High, Numeric Pain Rating Scale-Highest pain rating; NRS-Low, Numeric Pain Rating Scale-Lowest pain rating; PCS, Pain Catastrophizing Scale; PHQ-9, Patient Health Questionnaire for Depressive symptoms.

Figure 2 and Table 4 describe paired *t*-test results for the whole sample and subgroups with clinically elevated versus lower catastrophizing (PCS ≥ 30, PCS < 30). Comparing baseline to FRP discharge scores and controlling for race/ethnicity, gender and age, our primary analysis showed clinically meaningful (COPM delta ≥2) and statistically significant improvement in functional performance and satisfaction-with-function scores, with a large effect size in the whole sample, the subgroup with PCS ≥30, and the subgroup with PCS <30.

**Table 4:** Paired *t*-test results of total sample, PCS score < 30, and PCS score > 30.

	Variable	Baseline mean (SD)	Mean diff. (SD)	Conf. interval	*t*	*p*	Cohen’s *d*
Total sample *N* = 428	COPM-PS	3.42 (1.17)	2.34 (1.77)	2.17–2.5	2,723	<0.001	1.51^+++^
	COPM-SS	2.63 (1.23)	2.44 (2.23)	2.14–2.59	27.05	<0.001	1.54^+++^
	PCS	28.08 (11.89)	−3.60 (7.85)	−4.23–−2.86	−9.40	<0.001	−0.28^+^
	NRS-high	8.61 (1.54)	−3.33 (3.38)	−3.65–−3.01	−20.34	<0.001	−1.25^+++^
	NRS-low	3.62 (2.3)	−0.79 (2.04)	−0.98–−0.59	−8.01	<0.001	−0.36^+^
	PHQ-9	12.38 (6.49)	−1.69 (3.77)	−2.05–−1.33	−9.2	<0.001	−0.25^+^
PCS <30 *N* = 241	COPM-PS	3.53 (1.14)	2.52 (1.73)	2.30–2.74	22.60	<0.001	1.70^+++^
	COPM-SS	2.69 (1.17)	3.03 (2.08)	2.76–3.29	22.50	<0.001	1.75^+++^
	PCS	19.43 (6.96)	−3.17 (6.79)	−4.0	7.25	<0.001	−0.40^+^
	NRS-high	8.33 (1.64)	−3.68 (3.34)	−3.2–−4.1	17.13	<0.001	−1.39^+++^
	NRS-low	3.00 (2.05)	−0.66 (2.00)	−0.40–−0.91	5.1	<0.001	−0.34^+^
	PHQ-9	9.51 (5.20)	−1.56 (3.26)	−1.97–−1.14	−7.41	<0.001	−0.29^+^
PCS ≥30 *N* = 187	COPM-PS	3.28 (1.21)	2.09 (1.80)	1.83–2.35	15.89	<0.001	1.32^+++^
	COPM-SS	2.57 (1.30)	2.50 (2.18)	2.19–2.82	15.89	<0.001	1.32^+++^
	PCS	39.24 (6.33)	−4.16 (9.03)	–5.46–−2.86	−6.30	<0.001	−0.44^+^
	NRS-high	9.00 (1.25)	−2.87 (3.37)	−3.36–2.39	−11.66	<0.001	−1.13^+++^
	NRS-low	4.43 (2.21)	−0.93 (2.07)	−1.23–−0.63	−6.17	<0.001	−0.42^+^
	PHQ-9	16.10 (6.10)	−1.87 (4.34)	−2.50–−1.24	−5.87	<0.001	−0.28^+^

COPM-PS, Canadian Occupational Performance Measure–Performance Score; COPM-SS, Canadian Occupational Performance Measure–Satisfaction Score; NRS-High, Numeric Pain Rating Scale–highest pain; NRS-Low, Numeric Pain Rating Scale–lowest pain; PCS, Pain Catastrophizing Scale; PHQ-9: Patient Health Questionnaire-9.

Cohen´s *d* Effect size: +, small (0.2–0.49); ++, moderate (0.5–0.79); +++, large (>0.8).

For functional performance, as measured by the COPM-PS, 49% of participants demonstrated clinically meaningful improvement with baseline PCS ≥30 and 68% of participants with baseline PCS <30 (CHI2 = 15.56, *p* < 0.001). For satisfaction with function, as measured by the COPM-SS, 57% of participants with baseline PCS ≥30 and 69% of those with baseline PCS score < 30 achieved clinically meaningful improvement (CHI2 = 7.42, *p* = 0.007).

Pain, as measured by NRS-high scores, improved by an average of-2.87 among participants with baseline PCS > 30 (CI −3.36 to −2.39, *p* < 0.001) and improved by an average of-3.68 in those with PCS < 30 (CI −3.2 to −41, *p* < 0.001).

### 3.3 Secondary outcome measures

As presented in Table 4 and Figure 2, analysis of secondary outcomes revealed significant improvement in depressive symptoms and pain catastrophizing from admission to discharge for the full sample and each PCS subgroup. PHQ-9 scores significantly decreased (R2 = 0.01, Coef = −1.94, *p* = 0.002, CI = −3.16–−0.72). Catastrophic thoughts, measured by the PCS, significantly decreased after FRP completion (R2 = 0.0.01, Coef = − 4.11, *p* = <0.001, CI = −6.52–−1.70) for both those presenting with high pain levels (R2 = 0.11, Coef = −1.28, *p* = <0.001, CI = −1.5–−1.0), and for those presenting with lower levels of pain (R2 = 0.06, Coef = −1, *p* = <0.001, CI = −1.33–−0.66).

### 3.4 Fixed effects model regressions

Table 5 describes the fixed effects model regressions. After controlling for possible confounders and assessing for effect modifiers, all participants improved regardless of their baseline PCS score in all primary and secondary outcome measures, except for NRS-min score in the higher catastrophizing group. For all primary and secondary outcome measures, FRP participants with a PCS < 30 at baseline showed greater improvement when compared to participants with a baseline PCS >30 in. The difference was statistically significant (*p* values from <0.001 to 0.034).

**Table 5:** Multivariate analysis: fixed effect regression models results.

Effect of satisfaction score (COPM-SS)				
Independent variable	Slope	Std. error	*T*-ratio	Prob.
Time	3.15	0.15	20.36	<0.001*
Individual differences	−0.00014	0.00004	−3.0	0.003*
PCS <30 at baseline	−0.07	0.17	−0.045	0.65
Time*PCS High	−1.28	0.18	−6.97	<0.001*
Constant = 2.46; R2 = 0.11; F-Ratio = 11.95; *p* < 0.001; SEE: 2.09; *N* = 428				

Effect of functional performance score (COPM-PS)				
Independent variable	Slope	Std. Error	*T*-ratio	Prob.
Time	2.61	0.13	19.96	<0.001*
Individual differences	0.0001	0.00004	−2.17	0.030*
Age	0.007	0.003	2.11	0.035*
PCS <30 at baseline	−0.23	0.14	1.58	0.11
Time*PCS_High	−0.84	0.20	−4.04	<0.001*
Constant = 187.84; R2 = 0.41; F-Ratio = 54.23; *p* < 0.001; SEE = 1.48; *N* = 428				

Effect of highest pain rating (NRS-High)				
Independent variable	Slope	Std. Error	*T*-ratio	Prob.
Time	−3.72	0.19	−18.72	<0.001*
Individual differences	0.00004	0.00006	13.92	<0.001*
Female	0.44	0.17	2.57	0.010*
Age	−0.012	0.005	−2.27	0.02*
Race				
Black	0.61	0.31	1.97	0.049*
Other	0.81	0.27	2.95	0.003*
PCS <30 at baseline	0.54	0.22	2.45	0.014*
Time*PCS High	1.81	0.31	3.7	<0.001*
Constant = −17,878 R2 = 0.48; F-Ratio = 73.60; *p* < 0.001; SEE: 2.25; *N* = 428				

Effect of lowest pain rating (NRS-Low)				
Independent variable	Slope	Std. Error	*T*-ratio	Prob.
Time	−0.69	0.17	−3.85	<0.001*
Individual differences	0.00002	0.00005	0.43	0.66
Age	−0.013	0.005	−2.55	0.01*
Race				
Black	1.29	0.29	4.36	<0.001*
Other	0.61	0.25	2.36	0.018*
PCS <30 at baseline	1.34	0.20	6.69	<0.001*
Time*PCS_High	0.033	0.28	0.12	0.90
Constant = −46.37 Adjusted R = 0.15; F-Ratio = 14.26; *p* < 0.001; SEE: 203; *N* = 428				

Effect of depressive symptoms (PHQ-9)				
Independent variable	Slope	Std. Error	*T*-ratio	Prob.
Time	−1.71	0.48	−3.55	<0.001*
Individual differences	0.0001	0.0001	0.69	0.48
Age	−0.076	0.01	−5.90	<0.001*
Race				
Latino	−2.04	0.93	−2.19	0.02*
PCS <30 at baseline	6.43	0.54	11.89	<0.001*
Time*PCS_High	1.64	0.77	2.13	0.034*
Constant = −204.14; R2 = 0.34; F-Ratio = 39.52; *p* < 0.001 SEE = 5.47; *N* = 428				

PCS, Pain Catastrophizing Scale; COPM-PS, Canadian Occupational Performance Measure, -Performance Score; COPM-SS, Canadian Occupational Performance Measure-Satisfaction Score; NRS-High, Numeric Pain Rating Scale (highest pain rating); NRS-Low, Numeric Pain Rating Scale (lowest pain rating); PHQ-9, Patient Health Questionnaire-9; SEE, Standard Error of the Estimate.

### 3.5 Cohort analysis

Cohort analysis was conducted using self-reported race/ethnicity, gender, and age. While all groups made significant improvements, participants who self-reported race/ethnicity as Black or Other showed significantly less improvement in pain scores (NRS-max *p* = 0.49, NRS-min *p* < 0.001) compared to those who self-reported as White (NRS-max *p* = 0.003, NRS-min = 0.018). There were no significant differences in all other outcome measures between these groups. Additionally, individuals who self-identified as Latino exhibited greater reduction in depressive symptoms compared to those who self-identified as White (*p* = 0.02). Regarding gender cohorts, women showed significantly less improvement in NRS-max pain scores than men. (p = 0.02). Older participants demonstrated greater improvements in pain, COPM-PS, and PHQ-9 scores when compared to younger participants (NRS-max pain slope − 0.12 per decade of life; *p*-values from <0.001 to 0.035).

### 3.6 Exploratory findings on opioids

Because the FRP does not focus on opioid management, staff members do not systematically collect this data, so many participants had incomplete opioid data. 124 with complete opioid data were on opioids at the time of FRP admission. 52% (*N* = 65) reduced their opioid consumption by discharge. Exploratory analysis showed no statistically significant differences in opioid consumption (*p* = >0.05) due to incomplete opioid data and dosing variability among participants.

## 4 Discussion

This study examined the impact of FRP participation on individuals with high-impact chronic pain who have high levels of pain catastrophizing as measured by the Pain Catastrophizing Scale. This vulnerable population’s maladaptive cognitions related to their pain experience have previously been found to correlate with poorer outcomes and reduced efficacy of numerous treatments (Angst et al., 2022; Darnall et al., 2014; Martel et al., 2013; Wertli et al., 2014), including pain rehabilitation programs (Bergbom et al., 2011; Moore et al., 2016). It is notable that participants in both catastrophizing subgroups (PCS < 30 and PCS ≥ 30) achieved clinically and statistically significant improvements in all primary and secondary outcome measures: functional performance, satisfaction with performance, pain intensity, catastrophizing scores and depressive symptoms (Kirsch, 2018; Murphy et al., 2011).

Although high PCS scores are widely considered to be a negative prognostic indicator, in our FRP the subgroup with clinically elevated catastrophizing improved. We attribute these positive outcomes to three domains: (1) Enhanced therapeutic encounters with cohesive neuroscience education positively reframing treatment expectations; (2) Inclusion of integrated behavioral health and occupational therapy disciplines that are often underrepresented in IPPs; and (3) Interweaving conventional and integrative health approaches to self-care. Overall, these results support the role of IPPs facilitating enriched therapeutic encounters for individuals experiencing refractory, functionally limiting chronic pain.

Functional Restoration Program participants have already undergone screening at the start of the program to rule out pain generators that require medical or surgical intervention. Through unified and discipline-specific interventions described above, the program reinforces active learning that their pain is not dangerous and need not prevent them from engaging in valued activities. This understanding can be applied clinically in the setting of an enriched therapeutic encounter. Staff provide a supportive environment, validate participants’ experiences, address fears and concerns, and empower participants to partner with staff in the therapeutic process. It is common for individuals with high-impact chronic pain to have had negative healthcare experiences, resulting in nocebo-related outcomes and low expectations for success. The FRP provides ample time for patient-clinician interaction, focusing on empowerment of the individual to manage pain, utilizing pain neuroscience research, and facilitating positive treatment expectations. Rolling admissions allow newer FRP participants to witness the experience of participants who are further along in their program. Within this group context, participants observe their peers’ progress over the course of the program, enhancing a positive additive placebo effect (Ezzatvar et al., 2024).

In addition to reframing pain perceptions and facilitating enhanced function, hands-on treatment and repeated visits are among contextual factors that provide a ritual effect—a phenomenon found to enhance musculoskeletal outcomes in a recent review of physiotherapy randomized controlled trials (Bonanno et al., 2024; Kaptchuk et al., 2020). Moreover, hands-on interventions seem to promote functional changes in brain activity in adults with and without pain (Bonanno et al., 2024; Kaptchuk et al., 2020). Placebos, hands-on interventions, and some pharmacological treatments share common biochemical pathways and may activate the same receptor pathways, suggesting possible interactions among contextual factors, therapeutic rituals, and the activation of endogenous analgesic systems (Frisaldi et al., 2015; Frisaldi et al., 2020).

Given the social–emotional impact of pain and the established efficacy of biopsychosocial models, the need to incorporate behavioral health treatment into IPPs is clear (Pain Management Best Practices Inter-Agency Task Force Report, n.d.). Cognitive-Behavioral Therapy is considered the gold standard psychological intervention for chronic pain (Ehde et al., 2014) with proven efficacy in reducing disability, emotional distress, and catastrophizing (Schütze et al., 2018). Mindfulness-based Acceptance and Commitment Therapy and behavioral medicine interventions such as biofeedback and clinical hypnosis have also shown promise (Hilton et al., 2017). Despite this, behavioral health is inconsistently represented on IPP teams. A shortage of behavioral health clinicians trained to treat chronic pain is a barrier to care and may result in limited psychological treatment (The Behavioral Health Care Workforce, n.d.). Further research is needed on the influence of behavioral health or Cognitive Behavioral Therapy-trained clinicians on PCS outcomes within an IPP.

Individuals with high-impact chronic pain often lead restrictive lifestyles, eliminating activities that can enhance endorphin production, mood, and quality of life. Occcupational therapists are trained to analyze all components of a task, the individual, and their environment to help their patients perform meaningful life tasks comfortably. Even though occupational therapists’ role in improving functional outcomes has been well identified and recommended for addressing pain management nationally and globally (Breeden and Rowe, 2022; Lagueux et al., 2018; Rexe et al., 2013), and enhancing function is a primary IPP goal, many IPPs do not consistently staff OTs.

All FRP participants are trained in non-pharmacological treatments, including mindfulness, yoga, tai chi, biofeedback, imagery, and additional integrative health techniques. Participants’ prior experiences, myofascial tension, and conditioned responses result in anticipatory fear of familiar movement. Engaging in new movement patterns through tai chi, yoga, or Feldenkrais training may circumvent these expectations as they discover new ways of moving that are fluid and comfortable. Combined with mindfulness practice and diaphragmatic breathing, FRP participants develop interoception skills, decrease guarding, and discover new ways to reconnect with their bodies and resume desired activities. Acupuncture and massage therapy have been shown to improve mood and quality of life in patients with chronic pain (Yin et al., 2017), but they are passive treatments. In contrast, FRP staff teach self-massage and self-acupressure, which are active modalities and therefore a more viable option for IPPs. There is evidence for standalone use of integrative health techniques for chronic pain (Holtzman and Beggs, 2013; Kong et al., 2016; Lin et al., 2017), and the inclusion of these techniques in interdisciplinary rehabilitation programs requires further study (Bruns et al., 2019). Because pain-related suffering is a multifactorial phenomenon for which reductionistic, single-intervention approaches are often ineffective, FRP staff seek treatment synergy among interdisciplinary rehabilitation, a self-efficacy-promoting treatment milieu, and holistic mind–body awareness.

An exploration of ethnicity and race in our sample showed that self-reported Black participants had a significantly higher mean PCS score at baseline 34.17(12.60) compared to self-reported White participants, 26.92 (11.03). Previous literature indicates that Black participants report more frequent and disabling pain compared to other ethnic groups (Garvick et al., 2023; Mossey, 2011) yet when seeking pain treatment, Black patients are more likely to be referred for urine tests and substance treatment and less likely to receive analgesia prescriptions or procedures to assess and treat pain (Hoffman et al., 2016; Kennel et al., 2019; Morden et al., 2021), reflecting persistent bias within our healthcare system. Black FRP participants demonstrated clinically and statistically significant improvements in all outcome measures, although pain severity improved significantly less (mean reduction −3.03) compared to White FRP participants (mean reduction −3.7). Examining previous studies on racial disparities in IPP outcomes, this is consistent with Merry et al.’s finding that post IPP, Black participants improved in pain interference and depressive symptoms but not pain severity (Merry et al., 2011). Hooten et al. (2012) however, found that Black IPP participants made significantly less improvement than White participants in pain, depressive symptoms, PCS, and pain interference measures, highlighting the need for further research on race and pain management.

This study had several limitations. First, the study design was retrospective, which allowed us to show strong correlations but limited our ability to establish efficacy as a randomized, controlled trial could. Second, it lacked a control group, which would have reduced confounding factors but was not feasible in this clinical setting. Third, our population had a higher proportion of White, female participants, which limits generalizability based on race and gender. Finally, a common challenge of conducting research in a clinical setting is incomplete data collection. Workflow logistics, insurance constraints, and underrepresentation of behavioral health impeded our ability to consistently obtain discharge PCS and PHQ-9 scores. Our intention-to-treat approach assigned all missing discharge datapoints as “no change,” which may underestimate the impact of the intervention. Our sensitivity analysis supports the intention-to-treat results and reflects statistically significant clinical improvements in function, pain catastrophizing, and depressive symptoms.

Our findings add to the strong existing literature on clinical benefits of comprehensive interdisciplinary pain programs; and yet, access is limited. Of 50.2 million chronic pain sufferers in the United States, only 2.6% have participated in a self-management pain program (Yong et al., 2022). In 2022, the U.S had 50 CARF-accredited chronic pain programs, and their numbers are declining (Turk, n.d.). In the wake of the coronavirus pandemic, telehealth services offer new ways to deliver interdisciplinary, specialized care to individuals who face travel-related barriers. Since April 2020 our FRP has functioned virtually and in-person, offering new opportunities for access and further research.

Referral to IPPs often occurs after patients have already had numerous unsuccessful single-discipline medical, procedural, or physical therapy interventions, which can contribute to higher pain catastrophizing, negative treatment expectations, and subsequent even poorer outcomes. This study invites re-examination of how pain catastrophizing scores are utilized. Based on our results, we propose that elevated PCS scores be used as a tool for early referral to an IPP rather than considered a poor prognostic factor or disqualifier from participation (Cook et al., 2023; Sherriff et al., 2022). Early referral to evidence-based care that addresses the multifactorial nature of pain has great potential to lower pain catastrophizing and reduce the impact of chronic pain.

## 5 Conclusion

This is the first study to examine the functional impact of a Functional Restoration Program combining integrative health approaches with pain rehabilitation for individuals with high-impact chronic pain and significantly elevated pain catastrophizing. We observed strong associations between program participation and key outcomes including functional performance, satisfaction with function, pain, and mood. Further research is needed to maximize the functional capacity of this population, including larger, prospective controlled trials investigating the effect of interdisciplinary pain program participation on function, pain, analgesic use; racial and other disparities; the impact of early referral; and virtual platforms in relation to outcomes. Our results show promise for interdisciplinary pain programs to improve function, alter maladaptive thought processes, and reduce depressive symptoms for all individuals with high-impact chronic pain, including those with high pain catastrophizing.

### Author’s note

The study was not preregistered with an analysis plan through any institutional registry.

## Data Availability

The raw data supporting the conclusions of this article will be made available by the authors, without undue reservation.
